# A framework for T cell assays

**DOI:** 10.18632/oncotarget.6181

**Published:** 2015-10-19

**Authors:** Cedrik M. Britten, Sjoerd H. van der Burg, Cécile Gouttefangeas

**Affiliations:** GSK, Stevenage, Hertfordshire, UK

**Keywords:** immunotherapy, T cell assays, harmonization, standards, Immunology and Microbiology Section, Immune response, Immunity

T cell assays have undergone fundamental changes during the last 20 years. Major technological advancements have occurred, mainly driven by the development of more powerful flow cytometers, as well as innovative fluorophores and reagents that have increased the number of markers to be assessed simultaneously. In addition, the context in which these assays are utilized has shifted: while they were initially tools for basic immunology, they meanwhile have become a central element of biomarker programs accompanying clinical testing. Along with this evolution, more attention had to be paid to assay robustness, quality assessment and control of assay performance over time. This is of utmost importance, as proficiency panel activities conducted over the last 10 years by the Immunoguiding Program of the Association for Cancer Immunotherapy (CIP) and the Cancer Immunotherapy Consortium (CIC) have shown that *in vitro* assays can be subject to considerable lab-to-lab variation [1]. We propose that T cell assays should be embedded in a framework of measures guaranteeing performance overtime. This framework would comprise 4 components, (1) assay harmonization, (2) use of reference samples as quality control reagents, (3) standardization of data analysis, and (4) structured reporting of data. Only if these conditions are met, immunoassays are suitable tools to guide the development of novel therapies.

In 2010, a working model for harmonizing flow cytometry in multicenter clinical trials was proposed for the first time [2]. The suggested recipe was a combination of standardized processes directed by standard operation procedures (SOPs), use of quality controlled reagents, and implementation of reference samples for performing data acquisition at the different sites. In addition, a central lab would supervise the validity of SOPs and reagents, while data generation and analysis could be either done at peripheral sites or centrally. These recommendations complement the published **harmonization guidelines** for improved control of assay variation generated by CIP and CIC that have shown to lead to a better control of major technical sources of assay variation [1].

More recently, our labs have developed TCR-engineered reference cell samples (TERS) as a novel **reference standard** to control immune assay performance over time [3]. We showed that TERS, based on the transfer of RNA coding for TCR alpha and beta chains into primary T lymphocytes, can be utilized in the most common T cell assays and across a variety of known viral and tumour-associated antigens. TERS are stable, work in the hands of multiple international investigators and across different assay protocols. TERS implementation includes (1) controlled manufacturing, (2) assay-specific cut-off definition, and (3) application of TERS in the daily routine, which needs to be adapted to the protocols as they run in a given laboratory. Importantly, TERS were also shown to sensitively detect unwanted outcomes driven by common sources of inter-assay variation such as low cell viability, low reagent quality, suboptimal hardware settings and false analysis of flow cytometry data. Since scalability of the TERS technology was limited by the fact that batches needed to be prepared at a central manufacturing hub, we have meanwhile developed a kit-based methodology that allows shipping of quality-controlled RNA with defined shelf-life together with a manual allowing generation of TERS batches at peripheral sites.

We have also utilized serially-diluted TERS to support the development and optimization of one of the first computer-based algorithms for automated flow cytometry data analysis of low-frequency antigen-specific T cells [4]. Technologic advancements in flow cytometry nowadays generate high-dimensional data sets that cannot be handled anymore by standard manual gating approaches. This is recalling the developments in the field of genomics in the 1990s, when labs needed to develop bioinformatics tools to handle large data sets. In the very next future, a typical working group generating complex cytometry data sets will need dedicated immunologists doing the wet bench work complemented by bioinformaticians and biostatisticians. We have recently published a list of existing bioinformatics tools for controlling, processing, analyzing and visualizing high-dimensional data sets [5]. Even data sets of limited complexity need a controlled analysis strategy, as shown for 5-color flow-cytometry [6].

A final component of our proposed framework is to follow **reporting standards** [7]. The Minimal Information About T cell Assays (MIATA) project provides a blueprint on how to report T cell experiments in a way that allows a reader to transparently capture information on all assay key variables in a structured manner. The MIATA reporting framework has been adopted by a series of immunology journals (www.miataproject.org). Harmonization guidelines and repositories for the reporting and submission of flow cytometry data have already been proposed by others, allowing meta-analysis and data mining of annotated flow cytometry datasets (http://www.mibbi.org and http://www.flowrepository.org).

In summary, international coordinated efforts conducted during the last decade have developed a framework for application of T cell assays. These efforts have identified specific sources of variation of cellular assays, have resulted in considerable technical advancements and have promoted standards on assay conduct within the clinical setting. With harmonization guidelines and reference samples for the assays, standards for data analysis and structured reporting of results, the field is now ready to take full advantage of complex T cell assays to guide the development of novel immunotherapeutics.
